# Mutation screening in genes known to be responsible for Retinitis Pigmentosa in 98 Small Han Chinese Families

**DOI:** 10.1038/s41598-017-00963-6

**Published:** 2017-05-16

**Authors:** Lulin Huang, Qi Zhang, Xin Huang, Chao Qu, Shi Ma, Yao Mao, Jiyun Yang, You Li, Yuanfeng Li, Chang Tan, Peiquan Zhao, Zhenglin Yang

**Affiliations:** 1 Sichuan Provincial Key Laboratory for Human Disease Gene Study and the Department of Laboratory Medicine, Sichuan Academy of Medical Sciences & Sichuan Provincial People’s Hospital, School of Medicine, University of Electronic Science and Technology of China, 32 the First Ring Road West 2, Chengdu, Sichuan 610072 China; 2 Institute of Chengdu Biology, and Sichuan Translational Medicine Hospital, Chinese Academy of Sciences, Chengdu, Sichuan China; 3 Center of Information in Biomedicine, University of Electronic Science and Technology of China, Chengdu, Sichuan China; 4 Department of Ophthalmology, XinHua Hospital Affiliated to Shanghai Jiao Tong University School of Medicine, 1665 Kong Jiang Road, Shanghai, 200092 China; 5 Department of Ophthalmology, Eye & ENT Hospital of Fudan University, Shanghai, China; 6 Department of Ophthalmology, Sichuan Academy of Medical Sciences & Sichuan Provincial People’s Hospital, School of Medicine, University of Electronic Science and Technology of China, 32 the First Ring Road West 2, Chengdu, Sichuan 610072 China

## Abstract

Retinitis pigmentosa (RP) is highly heterogeneous in both clinical and genetic fields. Accurate mutation screening is very beneficial in improving clinical diagnosis and gene-specific treatment of RP patients. The reason for the difficulties in genetic diagnosis of RP is that the ethnic-specific mutation databases that contain both clinical and genetic information are largely insufficient. In this study, we recruited 98 small Han Chinese families clinically diagnosed as RP, including of 22 dominant, 19 recessive, 52 sporadic, and five X-linked. We then used whole exome sequencing (WES) analysis to detect mutations in the genes known for RP in 101 samples from these 98 families. In total, we identified 57 potential pathogenic mutations in 40 of the 98 (41%) families in 22 known RP genes, including 45 novel mutations. We detected mutations in 13 of the 22 (59%) typical autosomal dominant families, 8 of the 19 (42%) typical autosomal recessive families, 16 of the 52 (31%) sporadic small families, and four of the five (80%) X-linked families. Our results extended the mutation spectrum of known RP genes in Han Chinese, thus making a contribution to RP gene diagnosis and the pathogenetic study of RP genes.

## Introduction

Retinitis pigmentosa (RP, OMIM#268,000) is caused by abnormalities of the photoreceptors (rods and cones) or the retinal pigment epithelium (RPE) of the retina, and results in progressive vision loss^1^. RP is an inherited degenerative eye disease that causes severe vision damage and often results in blindness^1^. Affected individuals may experience difficulties in light-to-dark and dark-to-light adaptation or night blindness at the early stage of RP. RP is likely the most common type of retinal dystrophy. The worldwide prevalence of non-syndromic RP is approximately 1 in 4000^2^. The prevalence of non-syndromic RP in China had been reported at 1 in 3800^3^.

RP exhibits autosomal dominant (adRP), autosomal recessive (arRP), or X-linked (xlRP) models. In very rare cases, the cause is a digenic pattern of inheritance. Non-systemic RP represents about 70–80% of all cases^4^. Autosomal dominant, autosomal recessive, and X-linked account for approximately 30–40%, 50–60%, and 5–15% respectively of patients with RP^2^. Approximately 30% are sporadic cases^4^, most of which may belong to the autosomal recessive inheritance group.

RP genetics are complicated and heterogeneous. To date, 27 autosomal dominant, 58 autosomal recessive, and three X-linked RP genes have been identified in the RetNet database (http://www.sph.uth.tmc.edu/retnet/). Among these genes, six—*BEST1*, *NR2E3*, *NRL*, *RHO*, *RP1*, and *RPE65—*can cause both autosomal dominant and autosomal recessive RP. In addition, mutations in several genes, including *ABCA4* ^5^, *PROM1* ^6^, *PRPH2* ^7^, *C8orf37* ^8^, and *PRPF31* ^9^, can cause both RP and macular degeneration.

Because of the highly genetic heterogeneity of RP, an accurate genetic diagnosis is needed to improve clinical diagnosis^10^. In recent years, whole exome sequencing (WES) has been used for the molecular diagnosis of Mendelian diseases^11^. Although similar studies in RP have been published in the last few years, most of these reports were focused on the Caucasian population. Published RP mutations in the Chinese population are rare in the Human Gene Mutation Database (HGMD, http://www.hgmd.org/) and the Online Mendelian Inheritance in Man (OMIM, http://omim.org/). Different populations may have different mutation spectra, which is very important in studying the origin and pathogenesis of heterogeneous diseases such as RP. In this study, we investigated the mutations of known RP genes in 101 patients in 98 small Han Chinese RP families, which is beneficial for RP gene diagnosis and the pathogenic study of RP.

## Methods

### Ethics statement

This project was approved by the Ethics Committee of the Hospital of the University of Electronic Science and Technology of China and Sichuan Provincial People’s Hospital, Chengdu, Sichuan, China; and the ethics committee of Xinhua Hospital, Shanghai Jiao Tong University, Shanghai, China. Written informed consent was obtained from all participants involved in this study. All experiments were performed in accordance with relevant guidelines and regulations, including any relevant details.

### Study subjects

Complete histories, pedigree analysis, and ophthalmic examinations were performed when sampling. Eye exams consisted of cycloplegic refractions, fixation testing, Snellen visual acuities (when possible), pupillary responses, slit lamp exams, dilated fundus exam by indirect ophthalmoscopy, retinal photography, and Goldmann visual field analyzer and Humphrey field analyzer testing (when possible).

Electroretinograms (ERGs) were performed according to International Society for Clinical Electrophysiology of Vision standards. Patients with a rod-specific b wave that was reduced or undetectable and a color fundus photo of the eye with intraretinal pigment migration were included in this study. The criteria for defining RP in the families were based on the probands’ descriptions of the features of their family members, such as poor vision and night blindness, and then confirmed by clinical examination. DNA samples were extracted from whole blood. The concentration of DNA was determined by using a Nano Drop spectrophotometer.

### WES experiments and data analysis

We sequenced 101 RP patients using the Illuminan Truseq Enrichment System Capture, following the manufacturer’s instructions on the Illumina HiSeq 2500 platform. The sequencing reads were mapped against UCSC hg19 (http://genome.ucsc.edu) by BWA (http://bio-bwa.sourceforge.net/). Individual sample single nucleotide polymorphisms (SNPs) and insertion or deletion events (indels) were detected by SAMTOOLS (http://samtools.sourceforge.net/). After generating initial single non-synonymous variant (SNV) calls, we performed further filtering to identify high-confidence variants that (i) had quality >Q30 and depth of ≥5× and (ii) were not located in the major histocompatibility complex homologous sequence.

WES data from 1000 Genomes (http://browser.1000genomes.org/index.html), dbSNP135-common, the ExAC database (http://exac.broadinstitute.org/), and unrelated individuals of 2020 in-house non-RP controls were used as reference data for variant filtering. Prediction of potential functional consequences of variants was conducted using SIFT and PROVEAN (http://sift.jcvi.org/www/SIFT_chr_coords_submit.html)^12^, and Polymorphism Phenotyping v2 (PolyPhen-2, http://genetics.bwh.harvard.edu/pph2/)^13^.

Autosomal recessive, autosomal dominant, X-linked, and digenic heredity models were all considered in this study. The mutations were filtered with the following multiple-step bioinformatics analysis: (1) the SNPs and short indels in the exome region were filtered against data from 1000 genome, dbSNP135-common, ExAC and unrelated individuals of 2020 in-house non-RP controls, removing minor allele frequency (MAF) values that were greater than 0.005 for recessive model and any frequency for dominant model; (2) excluded non-coding variants without altering splicing sites; (3) excluded the synonymous variants without altering splicing sites in the genes; (4) excluded missense variants predicted to be Neutral/Tolerated /Benign by PROVEAN, SIFT and Polyphen-2 simultaneously.

### Polymerase chain reaction and direct Sanger sequencing for variant confirmation

If pathogenic mutations were found, further Sanger validation and segregation were carried out. Primers were designed (Primer3, http://biotools.umassmed.edu/bioapps/primer3_www.cgi) to use polymerase chain reaction (PCR) amplification on the 400–500 bp region flanking the mutation. To ensure high-quality Sanger sequencing, the amplification was designed to have a boundary at least150 bp away from the mutation base. The amplification was then Sanger sequenced on an Applied BioSystems 3730 capillary sequencer (Waltham, MA, USA). The Sanger sequencing results were analyzed with Applied BioSystems’s Sequencer software. The samples of RP family members were also sequenced by Sanger sequencing to perform segregation analysis, and clinical reevaluation (if necessary) were carried out after mutations found. We defined variants as “compound heterozygous” in a patient when we detected the patient’s father and mother each carrying a heterozygous mutation or the direct relatives without RP only carrying a heterozygous mutation. Variants were excluded when the exactly same variants were detected in the relative who did not diagnosed with RP phenotype. When RP patients’ mutations were not detected in their biological parents, we defined these mutations as “*de* no*vo*”. We defined a variant as “novel” if it had not been reported in the literature or registered in HGMD and OMIM databases.

## Results

### Whole exome sequencing of 101 RP patients in 98 RP families

We recruited 98 families clinically diagnosed with RP, which included 22 adRP families, 19 arRP families, 52 sporadic small families, and five xlRP families. To obtain all the variants in the coding regions, we performed WES for 101 patients (two samples were sequenced in three families, and one sample was sequenced in each of the remaining 95 families), with an average of 9.6 Gb of sequence with 96× coverage of average throughput depth of exome target regions for each individual, leading to a total of 101 paired-end, base pair reads. The summary of the average output of each sample is shown in Supplementary Table 1.

Since RP is a rare Mendelian disease, variants with a frequency <0.5% for the recessive model^10^ and no frequency for the dominant model were kept for further consideration. We first searched for variants of the 77 known RP genes in the RetNet database of autosomal dominant, autosomal recessive, and X-linked RP genes. Then, to identify potential pathogenic mutations among rare variants in each patient, we searched for variants that matched the reported inheritance pattern of the respective genes: novel or previously reported heterozygous mutations in dominant RP genes, homozygous or compound heterozygous variants in recessive RP genes, and homozygous or heterozygous variants in X-linked RP genes. Missense variants predicted to be pathogenic by at least one predictor were kept for segregation analysis: deleterious by PROVEAN, damaging by SIFT or probably damaging by Polyphen-2. All identified potential pathogenic variants were then validated by Sanger sequencing within families. Segregation analysis was performed within family members.

### Putative pathogenic mutations in known RP genes

**Figure 1:**
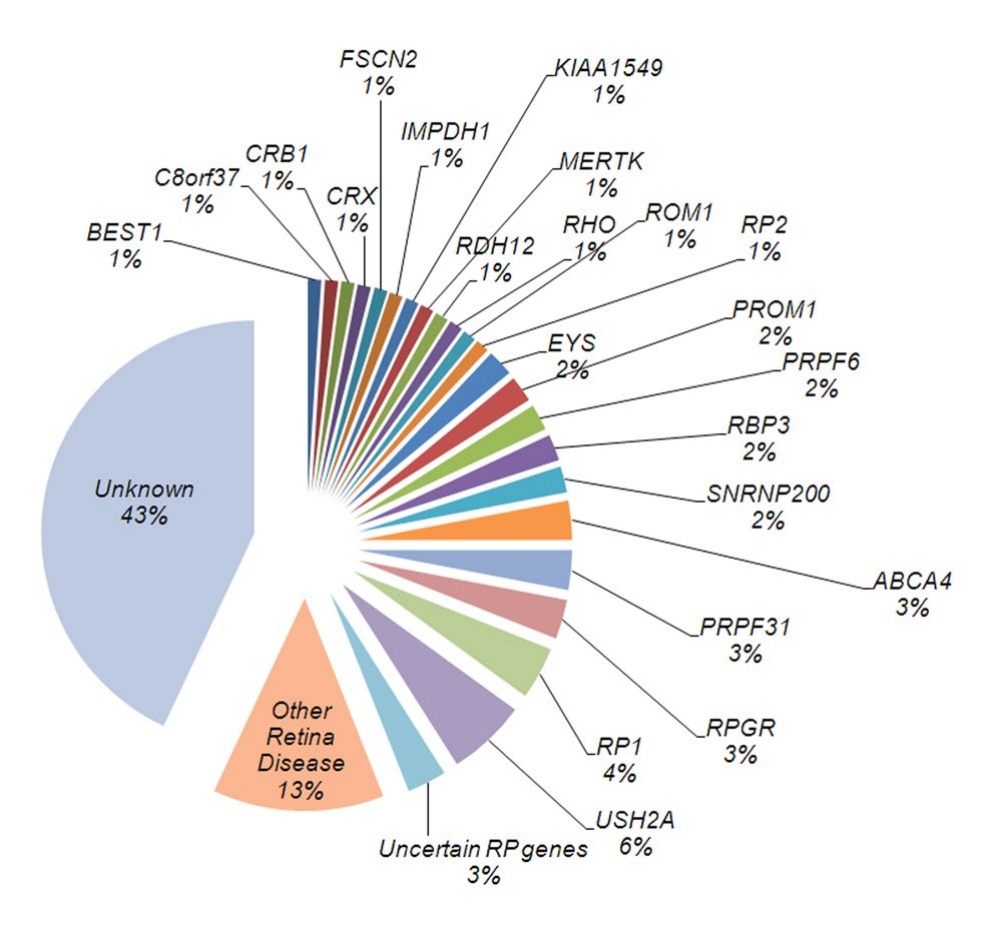
Mutation proportions of individual genes in the 98 small RP families. Mutation proportions of individual genes in the 98 small RP families were shown. The proportion of uncertain genes (with conserved amino acid prediction results and with low frequencies, Table 2), other retinal genes (mutations in other retinopathy genes, Table 3) and unknown genes were also shown.

We identified possible pathogenic mutations for 40 of the 98 (41%) families, which contained 57 mutations in 22 of the 77 known RP genes (Table 1, Fig. 1). The 57 mutations included 12 reported mutations: p.Asn247Ile^14^, p.Glu1122Lys^15, 16^, p.Ala1773Val^17^, and p.His55Arg^18^ in *ABCA4*; p.Arg526*^19^ in *CRB1*; p.Cyc2139Tyr^20^ in *EYS*; p.Gly122Asp^21^ in *CRX*; p.Asp311Asn^22^ in *IMPDH1*; p.Pro347Leu^23^ in *RHO*; p.Arg1653*^24^, c.8559-2A > G)^25^, and p.Cys934Trp^26^ in *USH2A* (Table 1). Thirteen of the 22 (59%) dominant families, eight of the 19 (42%) autosomal recessive families, 16 of the 52 (31%) of the sporadic small families, and four of the five (80%) of the X-linked families had identified mutations. A summary of the patients’ clinical diagnoses and presentations is shown in Supplementary Table 2.

**Table 1:** The 57 potential pathogenic mutations in 77 known RP genes in the 40 of the 98 small Han Chinese families with RP.

Gene	Inheritance model	Families	Mutation	Type	Change	PROVEAN	SIFT	Polyphon-2	Reported
*ABCA4*	sporadic	RP-047	c.6083C > T,p.Thr2028Ile	compound heterozygous	nonsynonymous	Deleterious	Damaging	probably damaging	Novel
*ABCA4*	sporadic	RP-047	c.740A > T,p.Asn247Ile	compound heterozygous	nonsynonymous	Deleterious	Damaging	probably damaging	Rivera *et al*.^14^
*ABCA4*	sporadic	RP-070	c.3364G > A,p.Glu1122Lys	compound heterozygous	nonsynonymous	Deleterious	Damaging	probably damaging	Lewis *et al*.^15^
*ABCA4*	sporadic	RP-070	c.1496G > A,p.Trp499*	compound heterozygous	stopgain	NA	NA	NA	Novel
*ABCA4*	sporadic	RP-134	c.5318C > T,p.Ala1773Val	compound heterozygous	nonsynonymous	Deleterious	Tolerated	probably damaging	Stenirri *et al*.^17^
*ABCA4*	sporadic	RP-134	c.164A > G,p.His55Arg	compound heterozygous	nonsynonymous	Deleterious	Damaging	probably damaging	Downs *et al*.^18^
*BEST1*	sporadic	RP-128	c.362G > C,p.Gly121Ala	homozygous	nonsynonymous	Deleterious	Tolerated	probably damaging	Novel
*RBP3*	sporadic	RP-042	c.3635C > T:p.Thr1212Ile	compound heterozygous	nonsynonymous	Neutral	Tolerated	benign	Novel
*RBP3*	sporadic	RP-042	c.2603T > C:p.Ile868Thr	compound heterozygous	nonsynonymous	Neutral	Damaging	possibly damaging	Novel
*RBP3*	sporadic	RP-156	c.787G > T:p.Ala263Ser	compound heterozygous	nonsynonymous	Neutral	Tolerated	possibly damaging	Novel
*RBP3*	sporadic	RP-156	c.97A > G:p.Lys33Glu	compound heterozygous	nonsynonymous	Neutral	Damaging	possibly damaging	Novel
*C8orf37*	recessive	RP-109	c.536A > G,p.Tyr179Cys	homozygous	nonsynonymous	Deleterious	Damaging	probably damaging	Novel
*CRB1*	sporadic	RP-052	c.1576C > T,p.Arg526*	compound heterozygous	stopgain	NA	NA	NA	Seong *et al*.^19^
*CRB1*	sporadic	RP-052	c.3442T > C,p.Cys1148Arg	compound heterozygous	nonsynonymous	Deleterious	Damaging	probably damaging	Novel
*CRX*	dominant	RP-046	c.365G > A,p.Gly122Asp	heterozygous	nonsynonymous	Neutral	Damaging	benign	Zernant *et al*.^21^
*EYS*	sporadic	RP-084	c.4245G > T,p.Gln1415His	compound heterozygous	nonsynonymous	Neutral	Damaging	benign	Novel
*EYS*	sporadic	RP-084	c.3489T > A,p.Asn1163Lys	compound heterozygous	nonsynonymous	Deleterious	Damaging	probably damaging	Novel
*EYS*	recessive	RP-097	c.6416G > A,p.Cys2139Tyr	homozygous	nonsynonymous	Deleterious	Damaging	probably damaging	Audo *et al*.^20^
*FSCN2*	dominant	RP-149	c.227G > A,p.Arg76His	heterozygous	nonsynonymous	Neutral	Damaging	probably damaging	Novel
*IMPDH1*	dominant	RP-121	c.931G > A,p.Asp311Asn	heterozygous	nonsynonymous	Deleterious	Damaging	probably damaging	Bowne *et al*.^22^
*KIAA1549*	sporadic	RP-001	c.5636C > T,p.Pro1879Leu	compound heterozygous	nonsynonymous	Deleterious	Damaging	NA	Novel
*KIAA1549*	sporadic	RP-001	c.4697G > A,p.Arg1566His	compound heterozygous	nonsynonymous	Deleterious	Damaging	probably damaging	Novel
*MERTK*	sporadic	RP-010	c.296_297delCA,p.Thr99Serfs*8	homozygous	frameshift_deletion	NA	NA	NA	Novel
*PROM1*	sporadic	RP-029	c.1984A > T,p.Lys662*	compound heterozygous	stopgain	NA	NA	NA	Novel
*PROM1*	sporadic	RP-029	c.1911 + 1G > A,-	compound heterozygous	splicing	NA	NA	NA	Novel
*PROM1*	recessive	RP-041	c.1078-2A > T,-	compound heterozygous	splicing	NA	NA	NA	Novel
*PROM1*	recessive	RP-041	c.748delA,p.Met250Trpfs*15	compound heterozygous	frameshift_deletion	NA	NA	NA	Novel
*PRPF31*	dominant	RP-035	c.1222C > T,p.Arg408Trp	heterozygous	nonsynonymous	Deleterious	Damaging	probably damaging	Novel
*PRPF31*	dominant	RP-098	c.1231_1232delCA,p.Gln411Glyfs*63	heterozygous	frameshift_deletion	NA	NA	NA	Novel
*PRPF31*	dominant	RP-126	c.1231_1232delCA,p.Gln411Glyfs*63	heterozygous	frameshift_deletion	NA	NA	NA	Novel
*PRPF6*	dominant	RP-068	c.1495G > A,p.Val499Met	heterozygous	nonsynonymous	Deleterious	Tolerated	probably damaging	Novel
*PRPF6*	dominant	RP-081	c.542C > T,p.Pro181Leu	heterozygous	nonsynonymous	Deleterious	Damaging	probably damaging	Novel
*RDH12*	dominant	RP-62	c.121G > T,p.Val41Leu	heterozygous	nonsynonymous	Neutral	Damaging	benign	Novel
*RHO*	dominant	RP-017	c.1040C > T,p.Pro347Leu	heterozygous	nonsynonymous	Deleterious	Damaging	probably damaging	Sohocki *et al*.^23^
*ROM1*	dominant	RP-111	c.117T > G,p.Ser39Arg	heterozygous	nonsynonymous	Neutral	Damaging	probably damaging	Novel
*RP1*	sporadic	RP-008	c.5764A > G,p.Thr1922Ala	heterozygous	nonsynonymous	Neutral	Damaging	benign	Novel
*RP1*	recessive	RP-012	c.788_790delTAA,p.Ile263del	homozygous	nonframeshift_deletion	NA	NA	NA	Novel
*RP1*	recessive	RP-096	c.1482C > G,p.Asn494Lys	compound heterozygous	nonsynonymous	Neutral	Damaging	possibly neutral	Novel
*RP1*	recessive	RP-096	c.2194C > T,p.Gln732*	compound heterozygous	stopgain	NA	NA	NA	Novel
*RP1*	sporadic	RP-153	c.5311T > C,p.Ser1771Pro	heterozygous	nonsynonymous	Deleterious	Damaging	probably damaging	Novel
*RP2*	X-linked	RP-170	c.445C > T,p.Gln149*	hemizygote	stopgain	NA	NA	NA	Novel
*RPGR*	X-linked	RP-027	c.284G > A,p.Glu95Glu	hemizygote	nonsynonymous	Deleterious	Damaging	probably damaging	Novel
*RPGR*	X-linked	RP-054	c.2236_2237delCT,p.Glu746Argfs*23	hemizygote	frameshift_deletion	NA	NA	NA	Novel
*RPGR*	X-linked	RP-138	c.1477delC,p.Gly494Glufs*7	hemizygote	frameshift_deletion	NA	NA	NA	Novel
*SNRNP200*	dominant	RP-083	c.3454C > T,p.Arg1152Cys	heterozygous	nonsynonymous	Deleterious	Damaging	probably damaging	Novel
*SNRNP200*	dominant	RP-62	c.6025C > T,p.Arg2009Cys	heterozygous	nonsynonymous	Deleterious	Damaging	probably damaging	Novel
*USH2A*	recessive	RP-028	c.12512T > G,p.Val4171Gly	compound heterozygous	nonsynonymous	Deleterious	Damaging	probably damaging	Novel
*USH2A*	recessive	RP-028	c.8559-2A > G),-	compound heterozygous	splicing	NA	NA	NA	Nakanishi *et al*.^25^
*USH2A*	recessive	RP-033	c.13151G > T,p.Gly4384Val	compound heterozygous	nonsynonymous	Deleterious	Damaging	probably damaging	Novel
*USH2A*	recessive	RP-033	c.6683T > A,p.Val2228Glu	compound heterozygous	nonsynonymous	Deleterious	Damaging	possibly deleterious	Novel
*USH2A*	sporadic	RP-063	c.4957C > T,p.Arg1653*	compound heterozygous	stopgain	NA	NA	NA	Dreyer *et al*.^24^
*USH2A*	sporadic	RP-063	c.2802T > G,p.Cys934Trp	compound heterozygous	nonsynonymous	Deleterious	Damaging	probably damaging	Xu *et al*.^26^
*USH2A*	sporadic	RP-076	c.8188C > T,p.Pro2730Ser	compound heterozygous	nonsynonymous	Deleterious	Damaging	probably damaging	Novel
*USH2A*	sporadic	RP-076	c.6326_6331delATTTAG,p.Asp2109_Leu2110del	compound heterozygous	splice-acceptor	NA	NA	NA	Novel
*USH2A*	recessive	RP-116	c.13621C > T,p.Gln4541*	compound heterozygous	stopgain	NA	NA	NA	Novel
*USH2A*	recessive	RP-116	c.12409A > T,p.Arg4137*	compound heterozygous	stopgain	NA	NA	NA	Novel
*USH2A*	sporadic	RP-166	c.6950G > A,p.Gly2317Asp	compound heterozygous	nonsynonymous	Deleterious	Damaging	probably damaging	Novel
*USH2A*	sporadic	RP-166	c.3407G > A,p.Ser1136Asn	compound heterozygous	nonsynonymous	Neutral	Damaging	probably damaging	Novel

The mutations were filtered with the following multiple-step bioinformatics analysis: 1) the SNPs and short indels in the exome region were filtered against data from 1000 genome, dbSNP135-common, ExAC and unrelated individuals of 2020 in-house non-RP controls, removing minor allele frequency (MAF) values that were greater than 0.005 for recessive model and any frequency for dominant model; 2) excluded non-coding variants without altering splicing sites; 3) excluded the synonymous variants without altering splicing sites in the genes; 4) excluded missense variants predicted to be Neutral/Tolerated /Benign by PROVEAN, SIFT and Polyphen-2.

In the 22 known RP genes, there were six families in which compound heterozygous mutations were detected in *USH2A* (6%) (Table 1, Fig. 1), which was the most frequently mutated gene in the families under study. They had all been diagnosed as simple RP patients before we found mutations. Among these six families, family RP-028 had exon 44:c.8559-2A > G^25^ and p.Val4171Gly mutations; family RP-033 had p.Gly4384Val and p.Val2228Glu mutations; family RP-116 had p.Gln4541* and p.Arg4137* mutations; patient RP-063 had p.Arg1653* and p.Cys934Trp^26^ mutations; patient RP-076 had c.6326_6331delATTTAG (p.Asp2109_Leu2110del) and p.Pro2730Ser mutations; and patient RP-166 had p.Ser1136Asn and p.Gly2317Asn mutations. After mutations were detected, we revisited and re-diagnosed patient RP-116 as Usher type II, while the other patients had no obvious hearing problems.

Mutations in *RP1* accounted for 4%; families RP-008 and RP-153 showed heterozygous mutations in the *RP1* gene (p.Thr1922Ala and p.Ser1771Pro respectively). No parents in RP-008 and RP-153 carried the mutations, and all biological parents were excluded as RPs by eye examination, suggesting that these two mutations were *de novo*. Patient RP-012 showed homozygous c.788_790delTAA and p.Ile263del mutations, and patient RP-096 showed compound heterozygous mutations of p.Gln732* and p.Asn494Lys.

*RPGR* accounted for 3% of all the probands. Three families displayed hemizygote mutations in the *RPGR* gene of which RP-054 and RP-138 showed frameshift deletion mutations p.Glu746Argfs*23 and p.Gly494Glufs*7 respectively.

*ABCA4* showed a 3% frequency in the investigated families. Patients RP-047, RP-070, and RP-134 showed compound heterozygous mutations in the *ABCA4* gene. For patient RP-047, p.Asn247Ile^14^ and p.Thr2028Ile mutations were detected. Patient RP-070 had p.Trp499* and p.Glu1122Lys^15, 16^ mutations, and patient RP-134 had p.Ala1773Val^17^ and p.His55Arg^18^ mutations; these two patients were reassessed as cases of Stargardt macular dystrophy (STGD).

**Figure 2:**
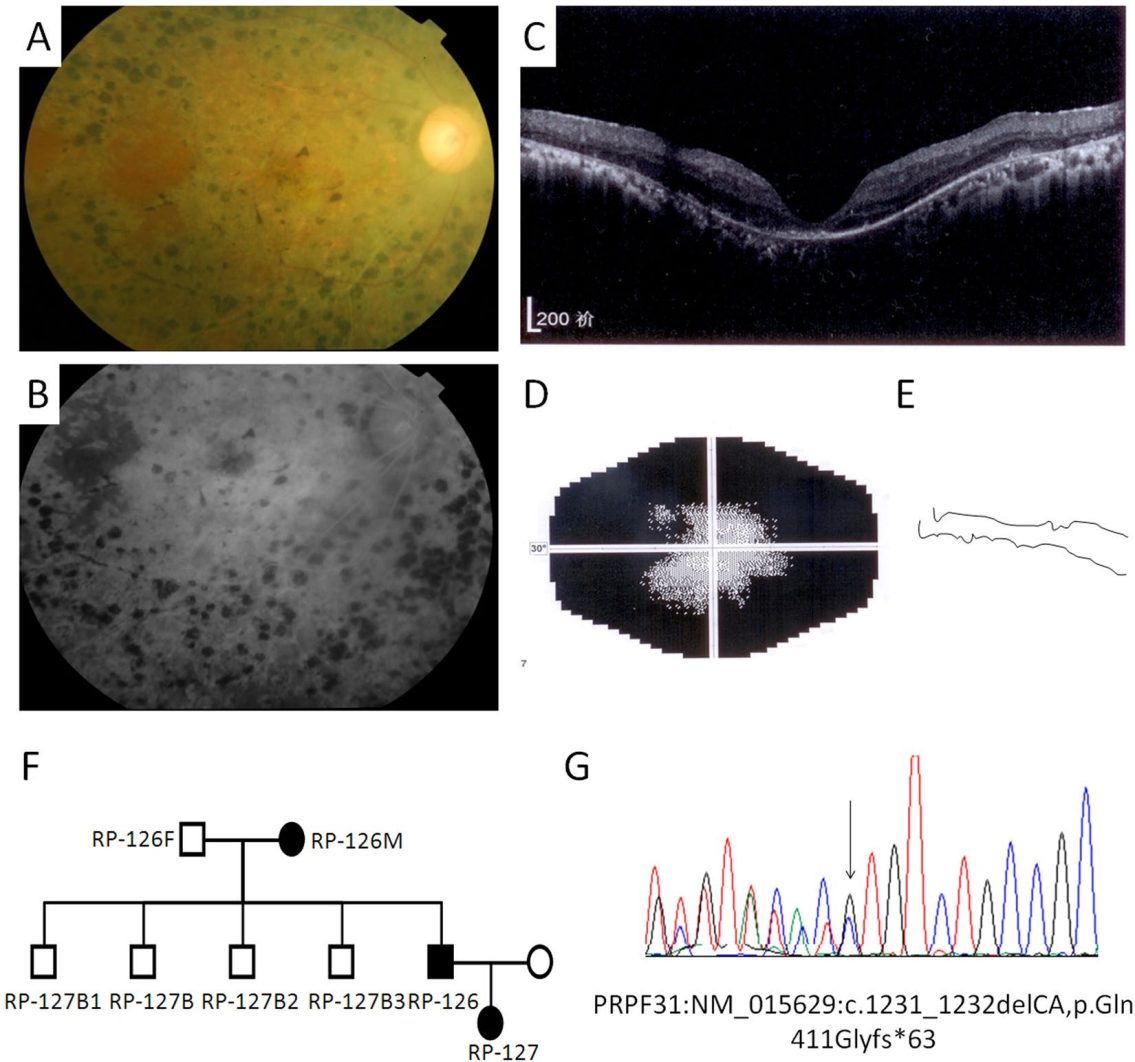
The phenotypes and mutation of patient RP-126 who carries a heterozygous mutation in the *PRPF31* gene. (**A**,**B**) Color fundus photograph (**A**) and while and black fundus photograph (**B**) of patient RP-126 shows a prominent multilobulated central atrophic maculopathy surrounded by concentric rings of black deposits. (**C**) Optical coherence tomography images of patient RP-126. (**D**) Vision field diagram of patient RP-126 shows the obvious vision loss (Humphrey automated threshold perimetry, Program 30-2). (**E**) ERG recording of A and B waves of patient RP-126 (30 µV/D, 25 ms/D). (**F**) Pedigree of RP-126 family. (**G**) The Sanger sequencing tracing of the mutation detected in the RP-126 family (*PRPF31*: NM_015629:c.1231_1232delCA, p.Gln411Glyfs*63).

*PRPF3* showed a 3% frequency in the investigated families. In *PRPF31*, the same mutation p.Gln411Glyfs*63 was identified in two families (RP-098 and RP-126). This mutation causes the protein to be pre-stopped at amino acid 474 (499 AAs for the wild-type PRPF31 protein), which might result in nonsense mediated decay. The clinical fundus pictures, DNA sequencing tracing, and analysis of amino acid conservation for this mutation are shown in Fig. 2. This frameshift deletion is believed to be the causative mutant of adRP.

Mutations in *PROM1*, *PRPF6*, *RDH12*, *EYS*, and *SNRNP200* showed a 2% frequency in the families studied. The mutations in the rest of the genes—*BEST1*, *CRB1*, *CRX*, *IMPDH1*, *MERTK*, *RHO*, *C8orf37*, *RP2*, *FSCN2*, and *ROM1*—showed only a 1% frequency in the 98 investigated families.

Of the 98 families, only RP-62 exhibited two novel mutations in two RP genes (*RDH12*, p.Val41Leu and *SNRNP200*, p.Arg2009Cys) (Table 1). Amino acid conservation analysis by PROVEAN, SIFT, and Polyphon-2 suggests that the p.Arg2009Cys is more likely to be the causative mutation in this family; however, we could not exclude the possibility of the two mutations working together to cause the disease phenotype in this family at this stage.

We may exclude the dominant mutations when we filter the variants in our control datasets if the non-RP controls show RP onset at the late adult stage after sampling. To avoid excess exclusion of possible causative mutations, we analyzed the heterozygous variants in sporadic RPs when the minor allele frequency was less than 0.5% in our in-house dataset; we then analyzed the amino acid conservation feature by using the PROVEAN and SIFT prediction methods. Next, we looked up the detected variants’ frequencies in the ExAC database of 60,706 unrelated individuals. We found three variants in *PRPF4* and *RDH12* genes with conserved amino acid prediction results and low frequencies (Table 2); these three variants may also be causative.

**Table 2:** Three candidate mutations with minor allele frequencies less than 0.001 in control samples in the 3of the 98 small Han Chinese families with RP.

Gene	Inheritance model	Sample	Mutation	Change	PROVEAN	SIFT	Polyphon-2	Allele Count in 2020 controls	Allele Count in 1000 Genomes Phase III	Allele Count in ExACdatabase	Allele Count East Asian	Allele Number East Asian
*PRPF4*	sporadic	RP-145	c.1541C > T:p.Thr514Ile	Nonsynonymous	Deleterious	Damaging	Probably damaging	2	0	0	0	8640
*RDH12*	sporadic	RP-050	c.940C > T:p.Arg314Trp	Nonsynonymous	Deleterious	Damaging	Probably damaging	2	0	2	0	8612
*RDH12*	sporadic	RP-060	c.437T > A:p.Val146Asp	Nonsynonymous	Deleterious	Damaging	Probably damaging	2	0	0	0	8612

The mutations were filtered with the following multiple-step bioinformatics analysis: 1) the SNPs and short indels in the exome region were filtered against data from 1000 genome, dbSNP135-common, ExAC and unrelated individuals of 2020 in-house non-RP controls, removing minor allele frequency (MAF) values that were greater than 0.001; 2) excluded non-coding variants without altering splicing sites; 3) excluded the synonymous variants without altering splicing sites in the genes; 4) excluded missense variants predicted to be Neutral/Tolerated /Benign by PROVEAN, SIFT and Polyphen-2.

We also analyzed the non-synonymous variants’ spectrum in the 77 genes known to be responsible for RP in the 101 RP samples and the 2020 in-house controls and found that about 30% of the known RP genes showed a conserved feature because very few non-synonymous variants could be detected either in RPs or in controls, including *PRPF3*, *TOPORS*, *PRCD*, *ZNF513*, *RP2*, *PDE6G*, *DHX38*, *ARL6*, *CRX*, *IDH3B*, *LRAT*, *SNRNP200*, *IMPDH1*, *PRPF6*, *PRPF31*, *GUCA1B*, *ARL2BP*, *PRPF8*, *NRL*, and *CLRN1*. In our in-house control dataset, the average count of non-synonymous variants of the RP genes was about 73 (3.9 variants per 100 sample); however, the RP genes listed above showed less than three non-synonymous variant counts in all samples (0.15 variants per 100 sampled). These results suggest that these genes may play very basic roles in human health in addition to their roles in the causation of RP.

### Mutations in other retinal disease genes

RP is one of the most commonly inherited retinopathy diseases and is highly heterogeneous. Mutations in other retinopathy genes might also cause the RP phenotype. Thus, we searched for mutations in another 161 retinopathy-related genes in the RetNet database. We detected eight genes that showed mutations in 11 sporadic samples (Table 3, Fig. 1). Mutations in seven genes (*KCNV2*, *HMCN1*, *CYP4V2*, *COL11A1*, *CAPN5*, *CACNA1F*, and *ADAMTS18*) typically cause only simple symptoms of RP, while patients with mutations in *IFT140* showed RP plus cataract (onset at 7 years of age). Three mutations in *CYP4V2* were detected in three samples (p.Trp340*, c.1091-2A > G^27^ (splicing), and p.Lys376Glu). RP-064 and RP-131 were detected as having the same compound heterozygous mutations (p.Trp340* and c.1091-2A > G)^27^. *CYP4V2* gene encodes a polypeptide of cytochrome P450 hemethiolate protein superfamily involved in oxidizing substrates in the metabolic pathway^28^. Mutations in this gene can also cause Bietti crystalline corneoretinal dystrophy in the recessive inheritance model^29^. Typical features of this disease include crystals scattered over the fundus, degeneration of the retina, and sclerosis of the choroid vessels, ultimately causing night blindness and vision loss^30^. Although the same mutations were detected in the present study in sporadic patients RP-064 and RP-131, both of whom were 25 years of age, different phenotypes were observed. Patient RP-064 was diagnosed with a syndrome causing lens opacity in both eyes. This patient developed the mottled appearance of the RPE caused by bone spicule formation, a waxy appearance of the optic nerve, and thin-walled blood vessels in the retina in both eyes. However, patient RP-131 showed only bone spicule formation in the retinas of both eyes, similar to another patient, RP-074, who had a *CYP4V2* p.Lys376Glu mutation. No syndrome was observed in either of these patients. Collectively, mutations in these genes may cause the heterogeneity in the clinical phenotypes of eye diseases.

**Table 3:** Fourteen potential mutations from 161 genes associated with other forms of retinopathy in the RetNet database in the 11 of the 98 small Han Chinese families with RP.

Gene	Previously reported disease	Inheritance model	Families	Mutation	Type	Change	PROVEAN	SIFT	Polyphon-2	Repodrted
*ADAMTS18*	Syndromic/systemic diseases with retinopathy	sporadic	RP-071	c.1985C > G,p.Pro662Arg	compound heterozygous	nonsynonymous	Deleterious	Tolerated	Benign	Novel
*ADAMTS18*	Syndromic/systemic diseases with retinopathy	sporadic	RP-071	c.113G > A,p.Cys38Tyr	compound heterozygous	nonsynonymous	Neutral	Damaging	Benign	Novel
*CACNA1F*	Other retinopathy	sporadic	RP-043	c.3094G > A,p.Glu1032Lys	homozygous	nonsynonymous	Deleterious	Tolerated	Possibly Damaging	Novel
*CAPN5*	Other retinopathy	sporadic	RP-107	c.1150T > G,p.Phe384Val	heterozygous	nonsynonymous	Deleterious	Damaging	Possibly Damaging	Novel
*CAPN5*	Other retinopathy	sporadic	RP-078	c.1150T > G,p.Phe384Val	heterozygous	nonsynonymous	Deleterious	Damaging	Possibly Damaging	Novel
*COL11A1*	Stickler syndrome /Marshall syndrome	sporadic	RP-009	c.1699C > A,p.Pro567Thr	heterozygous	nonsynonymous	Deleterious	Damaging	Possibly Damaging	Novel
*HMCN1*	Macular degeneration	sporadic	RP-056	c.10502C > T,p.Ser3501Leu	heterozygous	nonsynonymous	Deleterious	Damaging	Possibly Damaging	Novel
*HMCN1*	Macular degeneration	sporadic	RP-009	c.10682C > T,p.Thr3561Ile	heterozygous	nonsynonymous	Deleterious	Damaging	Benign	Novel
*IFT140*	Short-Rib Thoracic Dysplasia 9	sporadic	RP-024	c.3788C > T,p.Prp1263Leu	compound heterozygous	nonsynonymous	Deleterious	Damaging	Possibly Damaging	Novel
*IFT140*	Short-Rib Thoracic Dysplasia 9	sporadic	RP-024	c.1727G > T,p.Arg576Leu	compound heterozygous	nonsynonymous	Deleterious	Damaging	Possibly Damaging	Novel
*KCNV2*	Cone or cone-rod dystrophy	sporadic	RP-135	c.1074G > C,p.Glu358Asp	compound heterozygous	nonsynonymous	Neutral	Damaging	Benign	Novel
*KCNV2*	Cone or cone-rod dystrophy	sporadic	RP-135	c.1196C > T,p.Ala399Val	compound heterozygous	nonsynonymous	Deleterious	Damaging	Possibly Damaging	Novel
*CYP4V2*	Bietti crystalline corneoretinal dystrophy	sporadic	RP-131	c.1020G > A,p.Trp340*	compound heterozygous	stopgain	NA	NA	Na	Novel
*CYP4V2*	Bietti crystalline corneoretinal dystrophy	sporadic	RP-131	c.1091-2A > G,-	compound heterozygous	splicing	NA	NA	Na	Wang *et al*.^27^
*CYP4V2*	Bietti crystalline corneoretinal dystrophy	sporadic	RP-064	c.1020G > A,p.Trp340*	compound heterozygous	stopgain	NA	NA	Na	Novel
*CYP4V2*	Bietti crystalline corneoretinal dystrophy	sporadic	RP-064	c.1091-2A > G)	compound heterozygous	splicing	NA	NA	Na	Wang *et al*.^27^
*CYP4V2*	Bietti crystalline corneoretinal dystrophy	sporadic	RP-074	c.1126A > G,p.Lys376Glu	homozygous	nonsynonymous	Deleterious	Tolerated	Possibly Damaging	Novel

The mutations were filtered with the following multiple-step bioinformatics analysis: (1) the SNPs and short indels in the exome region were filtered against data from 1000 genome, dbSNP135-common, ExAC and unrelated individuals of 2020 in-house non-RP controls, removing minor allele frequency (MAF) values that were greater than 0.005 for recessive model and any frequency for dominant model; (2) excluded non-coding variants without altering splicing sites; (3) excluded the synonymous variants without altering splicing sites in the genes; 4) excluded missense variants predicted to be Neutral/Tolerated /Benign by PROVEAN, SIFT and Polyphen-2.

## Discussion

RP is the term given to a set of hereditary retinal diseases that feature degeneration of rod and conephotoreceptors^2^; these diseases typically present with poor night vision (due to rod dysfunction) in early or middle life. The condition of RP patients progresses to the loss of the mid-peripheral field of vision, which gradually extends and causes a small central island of vision due to the preservation of macular cones^2^. RP is one of the two main causes of blindness in those aged 20 to 64^31^. RP is a highly heterogeneous genetic disease. So far, mutations in 77 genes that can cause RP have been found in the RetNet database, and the number is increasing. In our WES data of 98 small RP families, we detected mutations in the genes known for RP in 40 (40.8%) families. It appears that the detected mutation rate might be higher among the larger pedigrees. In the present study, we detected mutations in known RP genes in 59% of the dominant inheritance families, 42% of the recessive families, 80% of the X-linked families, and 31% of the sporadic small families. This mutation detection rate is consistent with previous research on a Chinese RP cohort^32^. Most of the genes that cause inherited PR degeneration contribute to a small fraction of cases. Consistent with previous reports, the most common single genes that were found to cause RP in this study are *USH2A* ^2^, *RPGR* ^33^, *ABCA4* ^34^, and *RP1* ^35^. The frequency of the mutations that were detected in *RHO* was lower than in Caucasians (8–25%)^2, 4, 36^, but was similar to reports in Japanese (∼1%)^37^ and Indians (∼1%)^38^. Other genes caused only a small proportion of cases. The genetic diversity of RP presents challenges for clinical therapy, but molecular diagnosis would provide valuable information in gene replacement therapy^39, 40^.

In sporadic small families, which account for the vast majority of RP patients, it is difficult to confirm mutations, either for known RP genes or for the discovery of new RP genes, because co-segregation analysis cannot be performed. The possible reasons for the lower mutation rate detection of sporadic patients may include the fact that many genes responsible for sporadic RP have not yet been identified^41^, or the fact that non-genetic factors such as environmental phenocopies may easily occur in the case of sporadic RP^36, 42^. In our arRP families with detected known RP gene mutations in China, half were homozygotes, and the rest were compound heterozygous mutations. In sporadic patients, most were compound heterozygous mutations, which may be related to rarity of the phenomenon of close-relative marriages among the Han Chinese in China.

In this study, we used the WES method to examine the genetic etiology of RPs. We identified 57potential mutations in known RP genes, among which 12 are recurrent mutations and 45 are mutations first reported in this study. It is difficult to confirm a new missense mutation to be pathogenetic without *in vivo* and *in vitro* functional studies such as knock-in in animal models, a task that is beyond the scope of this study. Here, a multistep bioinformatics analysis strategy was utilized to systematically identify the putative pathogenic mutations for each of the 98 families (see method section). Our results extended the mutation spectrum of known RP genes, which will be beneficial for molecular RP diagnosis in the future. Our experience in demonstrating the high diagnostic yield by WES, coupled with its role in discovering etiologies, supports the use of WES in retinal disease practices. Although our diagnostic evaluation is a forward genetics approach, we hypothesize that it will be pivotal for future use in personalized genomic medicine and individualized RP treatments.

## Electronic supplementary material

Supplementary information
