# Supplementary material for: Mutation screening in genes known to be responsible for Retinitis Pigmentosa in 98 Small Han Chinese Families
Source: Sci Rep. 2017 May 16;7:1948. doi: 10.1038/s41598-017-00963-6 (PMC5434011; doi:10.1038/s41598-017-00963-6)
Supplement: Supplementary file 1 — Supplementary information [file 41598_2017_963_MOESM1_ESM.pdf]

Mutation screening in genes known to be responsible for Retinitis Pigmentosa in 98 Small Han Chinese Families

Lulin Huang[1,2,3*], Qi Zhang[4*],Xin Huang[5*], Chao Qu[6], Shi Ma[1,3,4],Yao Mao[1], Jiyun Yang[1,3],

You Li[1], Yuanfen Li[1], Chang Tan[1], Peiquan Zhao[4*], Zhenglin Yang[1,2,3 *]

Supplementary Table1 Summary of average output of sequencing data in individuals

Total reads	111,372,746
Total yield (bp)	9,648,647,346
Read length (bp)	101
Target regions (bp)	62,085,286
Average throughput depth of target regions	96
Initial mappable reads (mapped to human genome)	111,049,648
% Initial mappable reads (out of total reads)	99.70%
Non-redundant reads (de-duplicated by Picard tooles)	97,557,850
% Non-redundant reads (out of initial mappable reads)	87.90%
Non-redundant unique reads (uniquely mapped to human genome)	86,878,464
% Non-redundant reads (out of non-redundant reads)	89.10%
On-target reads (mapped to target regions)	61,139,313
% On-target reads (out of non-redundant unique reads)	70.40%
% Coverage of target regions (more than 1X)	96.30%
Number of on-target genotypes (more than 1X)	59,801,536
% Coverge of target regions (more than 10X)	92.10%
Number of on-target genotypes (more than 10X)	57,209,732
Mean read depth of target regions	80.30%
Number of SNPs	78,250
Number of coding SNPs	20,515
Number fo synonymous SNPs	10,673
Number of nonsynonymous SNPs	9,301
Number of Indels	8,072
Nmber of coding Indels	452

Supplementary Table 2 Summary of the patients' clinical diagnoses and presetation

			OD							OS		
**Patient**	**Gender**	**Age of onset**	**Vision**	**IOP （mmhg）**	**optic nerve head pale**	**Slender choriod vessels**	**Accumulation of bone spicules**	**Other symptoms**	**Vision**	**IOP （mmhg）**	**optic nerve head pale**	**Slender choriod vessels**
RP-001	female	Childhood	HM/BE	11	(+)	(+)	(+)		HM/20cm	9	（+）	（+）
RP-008	male	4	FC/10cm	18	（-）	（+）	（+）		FC/20cm	17	（-）	（+）
RP-009	male	Childhood	HM/20cm	11	(+)	(+)	(++)		HM/BE	11	（+）	（+）
RP-010	male	13	0.2	13	（-）	（+）	（+）		0.1	10	（-）	（+）
RP-012	male	Childhood	HM/BE	10	(+)	(+)	(++)		HM/BE	11	（+）	（+）
RP-015	male	27	LP	10	(+)	(+)	(++)	cataract	HM/10cm	12	（+）	（+）
RP-017	female	34	0.1	16	(+)	(+)	(++)		FC/30cm	14	（-）	（-）
RP-024	female	Childhood	HM	16	(+)	（+）	（+）	cataract	FC/20cm	19	NA	（+）
RP-027	male	Childhood	HM	13	（-）	（+）	(++)		0.3	16	（-）	（+）
RP-028	male	21	HM	14	（-）	（+）	（-）		HM	16	（-）	（+）
RP-029	male	27	LP	11	(-)	(+)	(++)		LP	15	（-）	（+）
RP-033	female	14	FC/40cm	15	（-）	（-）	（-）		FC/30cm	14	（-）	（-）
RP-035	female	20	0.25	11	（-）	（+）	（+）		0.2	10	（-）	（+）
RP-041	female	6	HM/BE	23	(-)	(+)	(++)		HM/BE	22	（-）	（+）
RP-042	male	Childhood	0.1(After correction)	18	(-)	(-)	(++)		0.12(After	15	（-）	（-）

									correction)			
RP-043	male	33	HM	11	（-）	（+）	（+）		LP	9	（-）	（+）
RP-046	male	10	HM	12	(-)	(-)	(++)		HM	10	（-）	（-）
RP-047	female	20	0.1	13	（-）	（-）	（-）		0.04	14	（-）	（-）
RP-048	female	13	FC/20cm(After correction)	15	(-)	(+)	(++)		FC/30cm(After correction)	13	（-）	（+）
RP-050	female	13	FC/30cm	11	(-)	(+)	(+++)		0.04	12	（-）	（+）
RP-052	male	Childhood	FC/40cm	16	（-）	（-）	（+）		FC/30cm	12	（-）	（-）
RP-054	male	20	HM	Tn	(-)	(+)	(++)		HM	Tn	（-）	（+）
RP-056	male	22	HM(After correction)	13	(-)	(+)	(++)		HM(After correction)	12	（-）	（+）
RP-060	female	28	FC/10cm	16	(-)	(+)	(++)		FC/20cm	19	（-）	（+）
RP-062	male	30	HM	14	(-)	(+)	(++)		LP	17	（-）	（+）
RP-063	male	30	LP	20	(-)	(+)	(++)		NLP	22	（+）	（+）
RP-064	female	25	HM/10cm	12	（+）	（+）	（+）	lens opacity	HM	10	（+）	（+）
RP-067	male	20	LP	11	（+）	（+）	（+）	lens opacity	LP	12	NA	NA
RP-068	female	26	LP	18	（+）	（+）	（+）	cataract	HM/BE	20	（+）	（+）
RP-070	female	4	0.01	8	（+）	（+）	（+++）	Stargardt disease	FC/30cm	9	（+）	（+）

RP-071	female	9	HM	8	（-）	（-）	（+)		0.25	10	（-）	（-）
RP-074	female	30	HM/20cm	13	(-)	(+)	(++)		HM	11	（-）	（+）
RP-076	male	24	HM	10	（+）	（+）	（++）	lens opacity	HM	8	（+）	（+）
RP-078	male	12	HM	13	（-）	（+）	（+）		HM/15cm	12	（-）	（+）
RP-081	male	38	0.4	16.3	（-）	（-）	（+）		0.2	14.3	（-）	（-）
RP-083	female	12	HM/BE	12	（-）	（+）	（+）		HM/BE	14	（-）	（+）
RP-084	female	27	HM	13	（+）	（+）	（++）		HM/10cm	11	（+）	（+）
RP-093	male	Childhood	0.02	12	（-）	（+）	（+）		0.02	12	（-）	（+）
RP-096	female	40	0.4	13	（-）	（++）	（+）		0.2	10	（-）	（++）
RP-097	male	10	0.5	13	（-）	（-）	（+）		0.12	13	（-）	（-）
RP-098	male	Childhood		0.01	（-）	（-）	（+）		0.08	14	（-）	（-）
RP-102	female	25	LP	18	（-）	（+）	（++）		LP	16	（-）	（+）
RP-107	male	Childhood	0.02	14	（-）	（+）	（++）		0.03	12	（-）	（+）
RP-109	female	15	LP	10	（-）	（+）	（++）		LP	13	（-）	（+）
RP-111	female	26	HM/BE	13	（-）	（+）	（++）		HM/BE	13	（-）	（+）
RP-116	male	14	FC/30cm	11	（+）	（+）	（++）	USHER	0.1	12	（+）	（+）
RP-121	female	Childhood	0.1	10	（-）	（+）	（+）		0.02	11	（-）	（+）
RP-126	male	Childhood	LP	12	（-）	（+）	（+）		LP	13	（-）	（+）
RP-128	male	26	HM	9	（-）	（+）	（+）		LP	8	（-）	（+）
RP-131	male	25	HM	10	（-）	（-）	（-、+）		HM	11	(-)	（-）

RP-134	male	15	0.01	15	（-）	（-）	（+）	Stargardt disease	HM	13	（-）	（-）
RP-135	male	35	LP	10	（-）	（+）	（+）		LP	10	（-）	（+）
RP-138	male	55	0.1	12	（-）	（+）	（+）		0.02	12	（-）	（+）
RP-145	male	congenital	FC/20cm	13	（-）	（-）	（+）		FC/20cm	14	（-）	（-）
RP-149	male	30	LP	20.5	（-）	（+）	（+）		HM	19	（-）	（+）
RP-153	female	Childhood	HM	7	（-）	（-）	（-、+）		FC/50cm	7	（-）	（-）
RP-156	male	Childhood	HM	10	（-）	（+）	（+）		HM	10	（-）	（+）
RP-166	male	28	LP	10	（-）	（+）	（++）		HM	13	（-）	（+）
RP-170	male	22	HM	15	（-）	（+）	（++）		HM	10	（-）	（+）
